# *“It’s the biggest not one-size-fits-all service I’ve ever worked in”*: the realities of delivering a ‘Complications of Excess Weight’ service for children and young people in England from a multidisciplinary team perspective

**DOI:** 10.1186/s12913-026-14899-z

**Published:** 2026-06-05

**Authors:** Rebecca A. Stone, Jordan Marwood, Paige Davies, James Nobles, Karen Coulman, Elysa Ioannou, Adam Martin, Veronica Swallow, Lucie Nield, Louisa Ells, Catherine Homer

**Affiliations:** 1 School of Health, Leeds Beckett University, Leeds, LS1 3HE UK; 2 School of Sport and Physical Activity, Sheffield Hallam University, Sheffield, S9 3TU UK; 3 Population Health Sciences, Bristol Medical School, University of Bristol, Bristol, BS8 1NU UK; 4 NIHR Bristol Biomedical Research Centre, University Hospitals Bristol and Weston NHS Foundation Trust and University of Bristol, Bristol, BS1 5DS UK; 5 Academic Unit of Health Economics, Leeds Institute of Health Sciences, School of Medicine, University of Leeds, Leeds, LS2 9NL UK; 6 School of Health & Social Care, College of Health, Wellbeing & Life Sciences, Sheffield Hallam University, Sheffield, S10 2BP UK; 7 School of Medicine and Population Health, University of Sheffield, Sheffield, S1 4DA UK

**Keywords:** Childhood, Children and young people, Obesity, Severe obesity, Weight management, Complications of excess weight, Healthcare, Patient centred care, Multidisciplinary teams, Qualitative

## Abstract

**Background:**

Thirty-eight specialist weight management services for children and young people (CYP) living with severe obesity are being piloted across England. These ‘Complications of Excess Weight’ (CEW) services aim to provide holistic, person-centred care to CYP, delivered by a multi-disciplinary team (MDT). The ENHANCE study (NIHR, 158453), a national evaluation of the CEW programme, sought to develop the evidence base and understanding of optimal models of care. This qualitative study aimed to explore the realities of delivering a CEW service from the perspective of MDT members.

**Methods:**

Online semi-structured one-to-one interviews (*N* = 28 MDT members) were conducted across seven purposefully sampled CEW services between April-June 2025. Interviews explored barriers and facilitators to delivery, perceptions of patient cohort, service demand, and how CEW fits within wider weight management systems. Data were transcribed and analysed using Framework Analysis.

**Results:**

Three overarching themes were identified: (1) *Achieving person-centred care in CEW services;* (2) *Navigating complexity in the CEW patient journey;* and (3) *The challenges of designing and delivering a pilot*. Participants described a complex patient group who required holistic, innovative approaches to care. Challenges included high service demand, a lack of appropriate step-down from CEW services, a lack of transitional care pathways into adult weight management for 16-18-year-olds, and uncertainty about sustainable long-term funding and staffing in the context of a pilot programme.

**Conclusions:**

A long-term commissioning plan would be needed to ensure stability and appropriate care for CEW services and the families they support. MDTs must be equipped with the skills to respond effectively to high levels of patient complexity, and gaps in the availability of less intensive services (step-down) and transitional care into adult weight management require urgent attention to ensure any positive medical and psychosocial improvements yielded from CEW are not lost.

**Supplementary Information:**

The online version contains supplementary material available at 10.1186/s12913-026-14899-z.

## Graphical Abstract

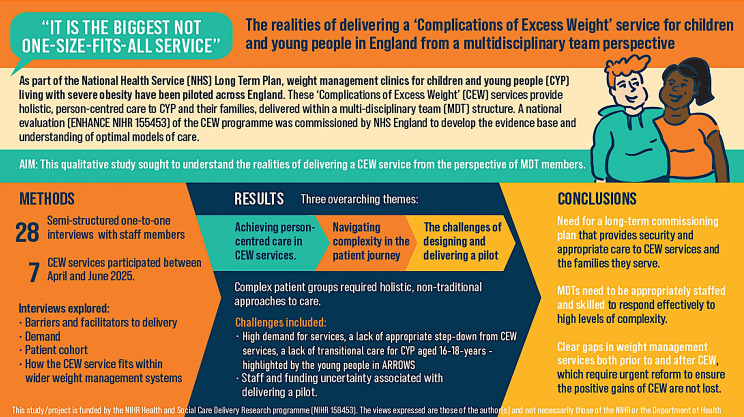

## Background

An estimated 8% of children and adolescents aged 5–19 years were living with obesity globally in 2022 (i.e., a body mass index (BMI) > 2 standard deviations above the median for their age/years and gender), compared with only 2% in 1990 [1]. In the UK, data from the National Child Measurement Programme of the 2024/25 academic year, showed 10.5% of children aged 4–5 years and 22.2% aged 10–11 years were living with obesity, including 2.9% and 5.6%, respectively, with severe obesity (BMI > 99.6th centile) [2]. However, this statistic may be an underestimate as children at this body weight are less likely to attend school [3]. In high-income countries, obesity is socially patterned, where in areas of deprivation obesity prevalence increases with deprivation quintile [4]. For example, in areas of higher deprivation in the UK, 30% of children aged 10–11 years are living with obesity, compared to 13% of children of the same age-group in the least deprived areas [5]. This highlights the growing prevalence in, and widening of inequalities regarding, obesity in CYP.

Severe obesity in childhood is widely recognised as a persistent health issue that tracks into adulthood [6, 7]. It is a key risk factor for developing many long term non-communicable diseases, such as cancer, Type 2 diabetes, and cardiovascular disease, all of which contribute to decreased life expectancy and elevated early mortality rates [8–10]. Indeed, recent modelling indicates that if a patient has a BMI z-score of 4 (i.e., severe obesity) by the age of 4-years and does not lose weight, they have a life expectancy of 39 years [11]. Childhood obesity is also linked to psychosocial consequences, such as lower self-esteem, lower academic attainment, and higher social exclusion [12]. Despite these significant impacts, weight management to support children and young people (CYP) living with severe obesity is limited [13, 14].

The NHS Long Term Plan committed to supporting 1,000 children per year, regarding complications related to severe obesity [15]. Subsequently, in 2021, NHS England commissioned the “Complications of Excess Weight” (CEW) pilot across England. This pilot initially funded 21 CEW services, increasing to 38 services by 2024. The primary aim of the programme was to provide person-centred, holistic treatment for CYP aged 2–17 years who were living with severe obesity and co-morbidities, with the goal of reducing the need for more invasive treatments later [16]. Each CEW service was given some autonomy to design and deliver their service tailored to the specific needs of their local population, which resulted in variability across different localities. Consequently, the ENHANCE study [17] was funded to provide a comprehensive understanding as to the make-up, effectiveness, and cost-effectiveness of the CEW programme nationally. In this paper, as part of the ENHANCE study, we aimed to explore the realities of delivering a CEW service from the perspective of multidisciplinary team (MDT) members from within CEW services.

## Materials and methods

This paper is reported according to COREQ guidelines for reporting qualitative research [18]. This study employed a qualitative design using semi-structured online interviews. Ethical approval was granted from Leeds Beckett University Ethics Committee (Ref: 128404) and Sheffield Hallam University Ethics Committee (Ref: 79269135) and is in accordance with the Declaration of Helsinki.

### Recruitment and sampling

Seven CEW services from across the UK were purposively sampled. Sampling was informed by geographical representation and responses to an adapted Standardised Reporting of Lifestyle Weight Management InTERventions (STAR-LITE) template [19] - findings presented elsewhere (Marwood et al., in prep; ENHANCE, [17] ). In short, the adapted STAR-LITE template systematically examined: a) the process of designing the CEW services; b) referral routes, capacity and waiting lists; c) the MDT composition, service delivery, pharmacotherapy, and complication management; d) discharge criteria and onward referral processes; and e) data management systems. Services were shortlisted for inclusion in the current study if they had detailed ‘innovative’ approaches to service delivery, including novel compositions of their MDT (i.e., not routinely seen across services, such as a family support worker) in their STAR-LITE response. Innovative approaches were determined using a systematic ranking criterion that was developed by the researchers. This consisted of eight criteria: composition of MDT; waiting list/ commissioned capacity; inclusion age; length of appointments; treatment interventions; Glucagon-like peptide-1 (GLP-1) use; discharge criteria; and transition services. Ten services were shortlisted and then pragmatically reduced to seven services in collaboration with NHS England (who have interests in service design/MDT availability) and the wider ENHANCE team. Additionally, within each selected CEW service, key staff were also purposefully sampled to ensure a range of MDT roles were represented. The lead clinicians of all seven services granted permission to recruit and conduct the interviews with the selected MDT members.

### Development of topic guide

The interview topic guide (Supplementary File 1) was co-produced with the wider ENHANCE team, which included academics, healthcare professionals and patient and public involvement. It explored each participant’s involvement in the CEW service and their experiences of the delivery of CEW, including perceptions of demand, descriptions of the patient cohort, and any adaptations made to the service. Experiences of working within an MDT and the innovative approach for why each service was selected to participate were also discussed. In addition, the topic guide explored barriers and facilitators to delivering the CEW services, and staff members’ perspectives on how the CEW service fits within wider weight management systems locally. Where relevant, topics such as safeguarding and onward or transition referrals were explored. The topic guide was piloted with staff based in a children’s specialist community weight management service to assess its utility and to gauge timings.

### Data collection

Individual interviews were conducted and recorded online using Microsoft Teams (April-June 2025). Written informed consent was obtained in advance of the interview and reconfirmed verbally during interview. Interviews were transcribed verbatim using Microsoft Team’s auto transcription feature into Microsoft Word and anonymised by a university approved provider, with final transcripts checked against the recordings for accuracy by RAS.

The research team involved in the interviewing and analysis process (RAS, JM, PD, JN) consisted of academics from varying career stages, with expertise in nutrition, public health, and behavioural science. They are skilled in conducting public health evaluations and informing evidence-based policy. This knowledge provides useful context for the conduct and interpretation of interviews, but we acknowledge the potential for bias in both the interpretation and representation of the data. Therefore, we include positionality statements to make explicit our roles and perspectives in relation to the research, which aligns with the principle of reflexivity [20] (Supplementary File 2).

### Data analysis

Transcripts were thematically analysed using inductive Framework Analysis (RAS and JM) following steps outlined in Gale et al. (2013) [21]. RAS and JM independently coded three transcripts and used these codes to jointly develop a working analytical framework, which was subsequently refined via application to three more transcripts. The final framework was used to analyse the remaining transcripts using NVivo, before generating a framework matrix using Microsoft Excel. Overall themes were generated from the framework matrix by examining connections between and within participants and codes. This analytical approach is often used for multidisciplinary healthcare research [21, 22] and is ideal for the analysis of semi-structured interview transcripts.

## Results

### Participants

**Fig. 1:**
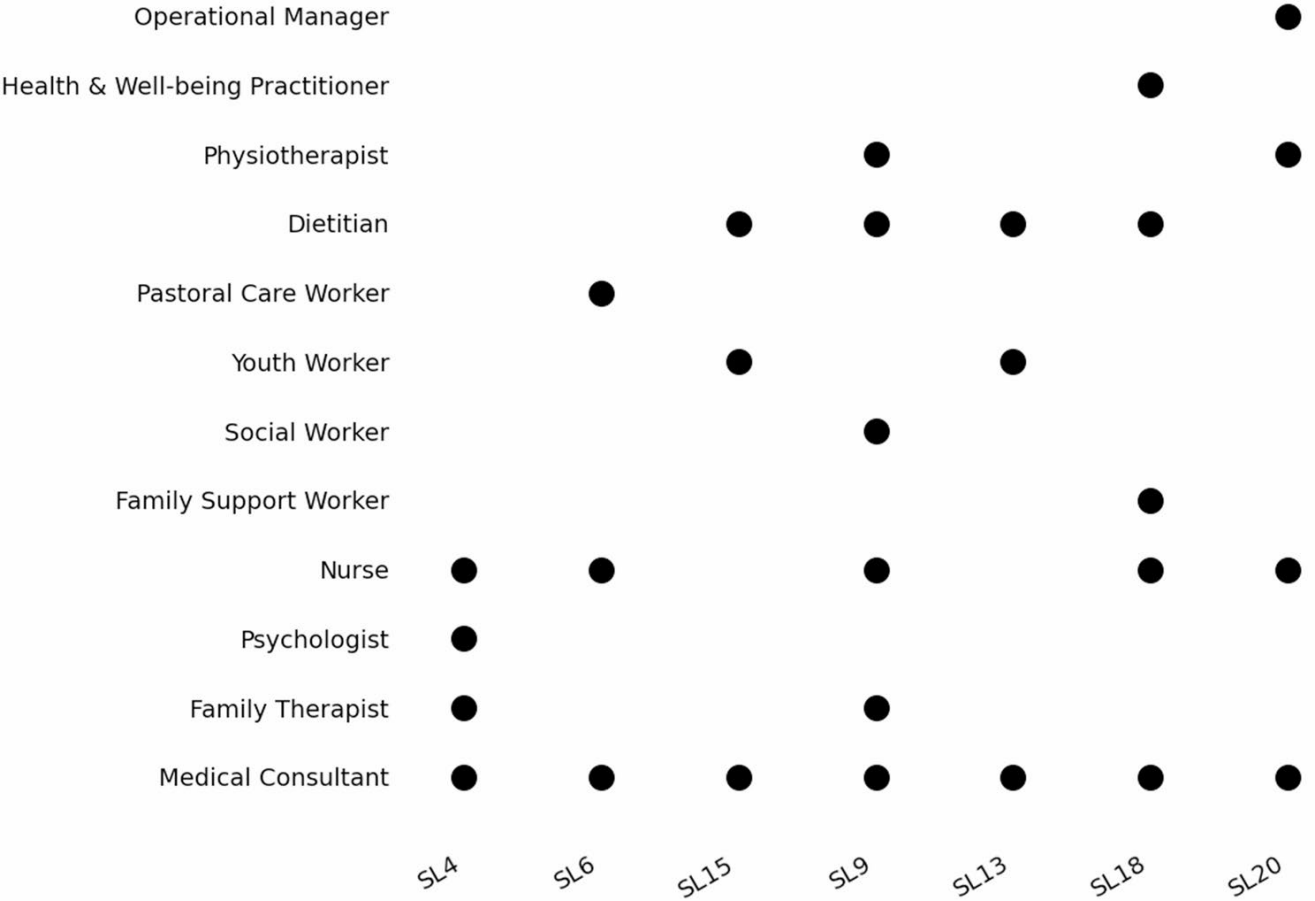
Dot matrix plot of the roles of MDT members (Y axis) interviewed from the seven CEW services (X axis). Note. Participant codes (e.g., ‘_2’) do not correspond to Fig. 1 and are not consistent across services to maintain anonymity. For example, SL4_2 and SL9_2 do not reflect the same MDT role. Roles listed on the Y axis do not exist in all seven services

In two services, the lead clinician requested that additional MDT members were invited to be interviewed 1:1 (SL9, *n* = 2; SL18, *n* = 1), and in three services, the lead clinician indicated that selected MDT members were no longer in post and were without replacements (SL6, *n* = 1; SL13, *n* = 1; SL15, *n* = 1). Therefore, in total, 28 staff members were invited to interview and all participated (see Fig. 1 for MDT role by service). Interviews were conducted by RAS (*n* = 18, 64%), PD (*n* = 8, 29%), or JN (*n* = 2, 7%), and lasted approximately 45 min (range: 29–67 min).

### Thematic findings

**Fig. 2:**
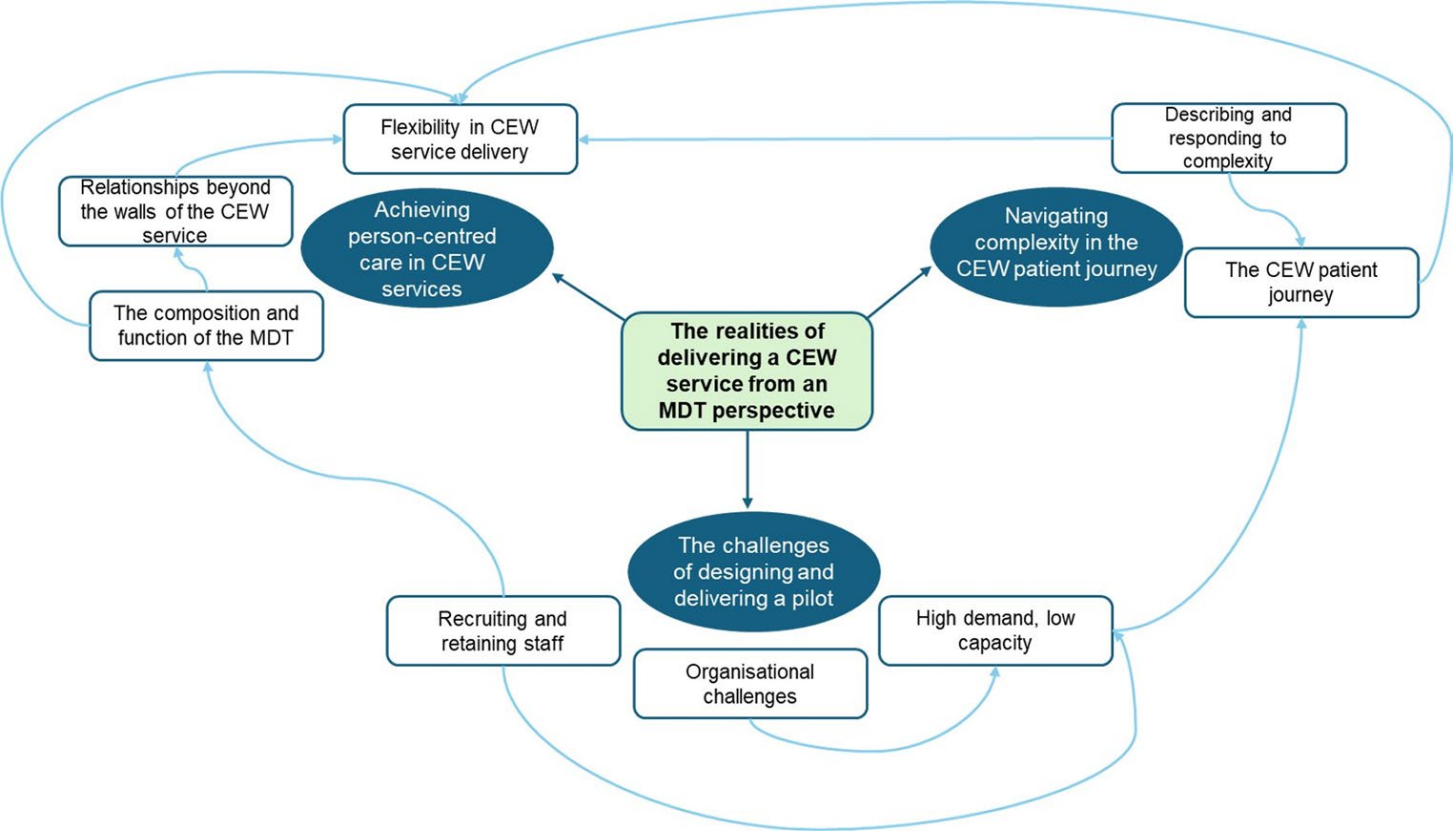
Thematic map of three major themes (ovals), eight sub-themes (rectangles), and relationships between sub-themes

Three major themes and eight sub-themes were generated in the analysis: (1) *Achieving person-centred care in CEW services*; (2) *Navigating complexity in the CEW patient journey*; and (3) *The challenges of designing and delivering a pilot* (Fig. 2).

The following section presents these themes, along with short example quotes. Further supporting quotations, over 4 lines long, can be found in Supplementary File 3.

#### Theme 1: Achieving person-centered care in CEW services

When exploring how CEW services were delivered, participants universally described how their service delivered flexible person-centered care. Participants reflected on how that care was delivered and suggested the composition and function of the MDT in CEW was central to this, as well as the importance of creating relationships external to CEW services, including through the wider CEW network and with other services (e.g., other hospital departments (e.g., Child and Adolescent Mental Health Services (CAMHS)), social services, and schools).

##### Subtheme 1.1: The composition and function of the MDT

Across the interviews, the composition of roles within the MDT varied. Participants referred to specific professions that were considered integral to delivering person-centred care in CEW, outside of core clinical roles (e.g., medical consultant, nurse, dietician). Given the level of complexity in the patient group (see Theme 2) and based on observations of efficiency in other services with a CEW social worker, many participants emphasised the need for a dedicated, “*embedded*” social worker within CEW, or a professional in the CEW MDT with a relevant skillset to support safeguarding. This was conceived to enable more effective and efficient safeguarding support, addressing perceived shortcomings in social services, and to reduce burden on other MDT members:

*The universal barrier to effective safeguarding is related to social services and their completely inconsistent approach to obesity as a risk factor. So*,* we [medics] spend a huge amount of time trying to explain to social services what the risks are* (SL15_1)

Family support workers were also seen as important to delivering person-centred care in CEW services due to their ability to holistically address family-based challenges experienced by CYP and their families. This was considered particularly important for younger children where autonomy is lower and family-level factors play an even stronger role in engagement with CEW services. In contrast to family support roles, some participants also highlighted the importance of including youth workers within the MDT to support adolescents who had greater agency. Youth workers were described as less authoritative and were felt to help de-medicalise CEW services for young people, which facilitated engagement:

*The youth worker is just cool; she’s brilliant at engaging CYP. They don’t see her as authority. She takes them out of the medical system very quickly and into community and other engagement projects* (SL15_1)

Notably, it appeared that services differed in the role title used for the youth worker role, with some adopting “health and wellbeing practitioner” instead. Nevertheless, the skill set required appeared to remain similar between roles:

*So*,* my role is called a health and well-being practitioner. Other services are called other things. Sometimes they’re youth workers*,* sometimes they’re psychological whatever. There’s lots of different titles for similar roles* (SL18_3)

Beyond professional expertise, participants emphasised that personal qualities and social capital were critical to the delivery of effective person-centred care in CEW services, particularly to *“open doors”* (SL18_4). In addition, the ability to build strong relationships with CYP was described as central to engagement, as well as having “*passion*” and “*genuine care”* given the “*ups and downs*” (SL15_3) of CEW services:

*It is that relationship building as well that’s been a really big thing that we have to*,* you have to be a certain kind of person to be able to kind of build those relationships with our young people and I think that’s a really big part of getting them to keep coming back if they feel like they’ve got a good relationship with* (SL9_5)

In addition, participants indicated that having previous experience of working with children with severe and complex obesity was crucial to anticipate and comprehend the level of patient complexity, and to provide care that is effective:

*I think I knew that*,* after years of working with this particular group*,* they’re very vulnerable and you need to think outside the box to be able to help them* (SL15_1)

Regarding the function of the MDT, participants highlighted the perceived benefits of having a dedicated office space, which facilitated team cohesion and improved morale:

*P: We’ve been lucky enough to have an actual office rather than people sitting in hot desking or anything like that. It used to be a cupboard*,* but now it’s at the office.*

*I: Has that made an impact?*

*P: Definitely*,* to have that space for the support of the team to be able to support each other and have a place*,* you know*,* that is theirs* (SL6_1)

Maintaining clear operational processes was also seen as key. Participants emphasised the importance of tracking actions discussed in MDT meetings to ensure efficiency and follow-through. One service used an online cloud spreadsheet to enable this:

*We use an online cloud spreadsheet so everyone can access at the same time*,* and it gets saved automatically*,* so no data is lost. So*,* once we’ve discussed it in MDT*,* we mark it so that doesn’t get transferred for the next week’s meeting* (SL9_1)

Importantly however, administrative support was seen as essential for enabling person-centred care in CEW services, and for supporting the level of data recording and reporting required by NHS England as part of the national evaluation (see Subtheme 1.2):

*But with that [person-centred care] comes the need to expand the admin that’s dedicated to make that run more smoothly essentially* (SL13_1)

##### Subtheme 1.2: Relationships beyond the walls of the CEW service

Participants often reflected on how their CEW service operated within the wider hospital system. They indicated that when other departments were in close proximity, the economies of scope, and spatial connectedness facilitated more efficient delivery of care:

*Most importantly*,* the other specialties would be able to address complications of obesity*,* they’re all here. So*,* I think for that point of view*,* it’s a really good environment because it’s one of the biggest Children’s Hospital in the country. You’ve got a lot of specialists here. It’s a huge hospital. They’ve got support*,* it’s very well connected* (SL20_1)

Participants’ perception of the utility of the national and regional CEW networks was largely positive. Almost all participants valued having a platform to share learning and exchange knowledge with their counterparts (see below), whereas for nurses, the network was reportedly not utilised as often due to difficulties in scheduling meetings.

*I’ve found the CEW network really helpful. I meet with other dietitians*,* because we’re often working in isolation*,* so that’s been handy to hear what they’re doing. We share information so we don’t have to reinvent the wheel if someone’s doing something else and they’re happy to share resources*,* so that’s been brilliant* (SL15_3)

Participants also acknowledged that CEW services can “*only do so much*” (SL15_2), and therefore engaging support from outside of CEW services (and the hospital) is important. Participants highlighted the value of building strong relationships with schools. In this illustrative quote, a relationship between a CEW service and school has enabled the CYP’s timetable to be adapted to better suit their needs, thereby increasing school attendance:

*So*,* we all link in with the schools as much as we can and get the timetables changed so that a young person that’s going in 20% of the week can actually go in 70% of the week and come out with their GCSEs [higher education qualifications in UK]* (SL13_3)

Similarly, participants noted the importance of building strong partnerships with local community services, explaining that these relationships helped CYP and their families access activities beyond CEW services, while also enabling CEW staff to understand “*what is out there*” (SL15_2) and signpost effectively according to CYP needs.

##### Subtheme 1.3: Flexibility in CEW service delivery

When thinking about how person-centred care is delivered in CEW services, participants often cited how they used non-traditional approaches to obesity (i.e., innovative in comparison to standard obesity care), which promoted a non-stigmatising/non-judgemental service. Participants indicated that these approaches encouraged CYP to be *“open and honest”* (SL6_2) about their food consumption, thereby facilitating holistic weight management support. Similarly, participants reflected on feedback received from CEW patients that celebrated CEW services’ ability to make the CYP feel heard, which was believed to contrast with previous experiences with healthcare:

*And I think some of the feedback we’ve had is that our team actually listen to what they’re saying. We understand where they’re coming from*,* we help them in a holistic way…* (SL6_2)

Participants emphasised the importance of flexibility in service delivery, describing CEW services as “*the biggest not one-size-fits-all service*” where “*you couldn’t follow a flow chart*” (SL6_2) to care. This flexibility was framed as a core component of the person-centred care delivered in CEW, enabling CYP to exercise autonomy in guiding their care. This approach was contrasted with other hospital services, such as diabetes or endocrinology, which were perceived as more prescriptive:

*The team have a flexible mindset more than anything else. We don’t have an agenda of what the CYP needs*,* they bring that to us*,* which is different to diabetes or endocrinology where you’re prescribing a set thing. But that doesn’t work for this group; you have to engage them by putting them at the forefront of every decision* (SL15_1)

In addition to flexibility in the *type* of support offered in response to CYP needs, participants also described flexibility in *how* support was delivered, including the mode of delivery (e.g., online, face to face, or group-based) and the scheduling of appointments. In the following illustrative quote, one respondent explained how expanding modes of delivery and opening hours of work helped accommodate CYP whose appointments would otherwise be during school hours:

*And the feedback that we got was flexibility was very key to getting them through the door. So*,* very quickly I realised we needed to get on all platforms and formats*,* so face to face*,* online*,* telephone*,* sometimes in groups*,* depending on the need. And also*,* to open up our hours of work as well* (SL18_4)

Similarly, this flexibility enabled CEW services to adapt their delivery in response to the presenting complexity of CYP. For example, participants often described making environmental adjustments to better meet the needs of neurodivergent CYP:

*We’ve got some children with more complex disabilities and needs*,* specifically around autism and ADHD. I contact parents*,* like*,* do you need to use our sensory room rather than one of our clinic rooms? Would you like me to meet you around the back of the hospital*,* and we can come in that way?* (SL9_3)

#### Theme 2: Navigating complexity in the CEW patient journey

This theme explores the complexity of the CEW patient group and how this influences patient engagement and their journey through the service, including preparation for CEW and transitions between step-down services and adult weight management services. It also captures the emotional impact on MDT staff working with this patient group.

##### Subtheme 2.1: Describing and responding to complexity

While the CEW programme targeted medical and psychosocial complications related to excess weight, participants observed additional, often unexpected and multifaceted complexities among attending patients:

*Never in my wildest dreams did I imagine some of these families. There is never a week where we don’t pick up on a new complexity*,* a new psychosocial risk factor* (SL6_2)

The range of complexities described included: neurodiversity and/or intellectual disabilities in children and/or caregivers, high levels of poverty and deprivation, low levels of literacy, mental health difficulties, geographical barriers, adverse childhood experiences in children and/or caregivers, housing issues, and language barriers. The impact of these complexities differed. For neurodiversity, participants highlighted how this could result in selective eating, rigidity around food choices, and the use of food as a behaviour management tool.

*I suppose with the children with Autism Spectrum Disorder (ASD) and attention deficit hyperactivity disorder (ADHD)*,* more ASD*,* the using of food as a behaviour management tool is*,* that’s one of our biggest areas we work with* (SL13_3)

For those from a lower socioeconomic group, participants highlighted how this impact included difficulties in attending meetings due to a lack of transport options, an inability to afford leisure activities and healthier food options, and a lack of access to cooking facilities for those families living in temporary accommodation:

*Well*,* I suppose because [county] has got some of the most deprived communities and the transport links aren’t great. So*,* getting to a big supermarket*,* often they’re reliant on little supermarkets*,* little sort of local shops*,* which are obviously going to be more expensive* (SL13_3)

Participants cited that there were high levels of safeguarding concerns observed in the CYP and families seen in CEW with social services involvement for many families. However, participants noted that there was often tension between social service teams and CEW due to lack of agreement on whether obesity is considered a safeguarding concern.

The complexity of the patient group necessitated adaptations in delivering and tailoring care. Participants discussed the need to upskill in their role using training and development opportunities to enable them to better care for patients, particularly those with neurodiversity. Others noted that it was difficult to adapt and deliver group materials due to different levels of comprehension within the patient group:

*Definitely we see a lot of our young people are autistic and have ADHD as well. And that comes with its own challenges and the need for us as professionals to be educated on ways to support them* (SL15_2)

Complexity in the patient group sometimes meant that families were unable to engage with CEW services due to high demands in other areas of their lives (e.g., housing issues). However, all services included within this sample noted that they accepted re-referrals so that families could access support when it was more suitable for them:

*Sometimes it’s probably too intense for them or they’ve got other things going on. I think some examples we’ve had are there’s been housing issues*,* school issues*,* there’s been maybe violence in the home*,* these sorts of things have happened*,* and it has just been we [the family] cannot give it the time that CEW needs now* (SL18_4)

##### Subtheme 2.2: The CEW patient journey

The patient journey begins with how CYP and families are prepared for entering the CEW service. Across the sample, approaches differed, with some services providing, or planning to provide, waitlist support, such as low-level healthy diet education, while other services did not. Participants noted that referrer communication quality was sometimes poor, where families were not made aware that they had been referred to CEW services or what the service entailed, and the subsequent perceived negative impacts this had on engagement with services:

*Referrers will say “I want you [CYP] to go to weight management [CEW]”*,* but don’t explain what weight management clinics [CEW] entail*,* and don’t explain properly why they’re [referrer] worried about the patient’s weight and the health impacts. So*,* the patient doesn’t have any buy-in* (SL13_1)

Participants often cited a general lack of ‘step-down’ services (i.e., less intensive than CEW, non-hospital support) to discharge CYP to. While some localities had paediatric Tier 2/behavioural weight management programmes, participants questioned their utility for CYP who had already received intensive support provided by CEW services. This was particularly evident in the following illustrative quote where a participant described a paucity of step-down services to support CYP’s discharge post-CEW:

*I often think we [CEW] represent this pyramid of what services should look like*,* and we are really setting ourselves up and crystallising what we need to do at the top*,* yet there’s absolutely nothing below us* (SL13_1)

Participants also noted that they found it difficult to “*keep on top of”* (SL9_2) the constantly changing weight management landscape (due to changes in commissioning and de-commissioning of Tier 2 services by local authorities), which was thought to hinder building connections with the right post-CEW services.

Staff also often cited a lack of transitional adult weight management services, which impacted CEW’s ability to discharge. This included an absence of, or oversubscribed, adult weight management services. Where these services did exist, participants noted that transitional care was not always optimal and amounted to “*transaction rather than transition”* (SL18_4). In some localities, there was a “*no man’s land*” (SL20_3) between paediatric and adult weight management services where CYP age out of CEW but are not yet old enough to attend adult weight management services. This was considered particularly problematic for the continuation of pharmacological weight management:

*They might come to us at 16 and then you’re considering do you start GLP-1s or not because you know there isn’t an adult service to transition into. You can’t keep them in paediatric care forever*,* so*,* does that mean they’re not getting the treatment they need*,* or they would have eligibility for*,* because there’s nowhere for them to go?* (SL9_5)

An associated issue was the length of intervention delivery. MDT members cited that their service operated flexibly, with a maximum intervention delivery length of two years. However, there was a consensus across interviews that it often takes significant time to engage families, gain trust, and establish rapport, as well as to address some of the complexities identified within the family before working on weight-loss and its associated complications. This issue, along with there being a lack of appropriate post-CEW services, was cited as the main reason for CYP remaining in services beyond the commissioned duration.

Finally, this subtheme captures the impact of complexity on how CYP engage with CEW services, and the emotional toll this can have on staff when supporting families in complex and challenging circumstances. This was particularly evident in discussions with participants around discharge and the difficulty of discharging CYP knowing that there will be little infrastructure to support them moving forward:

*… This job is relentless at times*,* and it can feel very much like you are drowning and not making any difference…You pour your heart and soul into these families*,* and you work with them for so long*,* and not being able to know that they’ve got a little bit of a safety net after us is hard* (SL6_2)

#### Theme 3: The challenges of designing and delivering a pilot service

Participants frequently referred to challenges experienced by CEW services, some of which were perceived to be due to the programme being at pilot stage. These included difficulties with recruitment and retention of staff, organisational challenges, and the resulting impact on MDT staff capacity to manage high demand for CEW services.

##### Subtheme 3.1: Recruiting and retaining staff

Several participants noted that they did not have a fully staffed MDT, often due to fixed-term, part-time and split-post contracts being “*not appealing*” (SL13_1), and the uncertainty associated with the future, longer-term funding of the CEW programme. Some participants described this as their “*biggest challenge*” (SL4_1) or as a “*total nightmare*” (SL15_1).

There were also difficulties in recruiting certain professionals to the MDT, such as psychologists, due to national shortages. Consequently, participants highlighted how some services had amended the role advertised to reflect that it fulfilled the requirement for psychological support without being a qualified psychologist, such as the use of a family therapist:

*When we were unsuccessful in getting a second psychologist*,* someone gave us the*,* the advice to look for a family therapist*,* so the next advert had psychologist or family therapist*,* and that has been tremendous. Although we weren’t looking for one*,* we thought the family therapist could ‘play the part’ of a psychologist* (SL4_1)

Participants highlighted how service delivery in CEW services were directly impacted by staffing issues. For example, one service had experienced difficulties in administering GLP-1s because of a lack of appropriate staff to run the necessary medication clinics, and the subsequent burden this placed on other MDT members to plug these gaps in care.

##### Subtheme 3.2: Organisational challenges

This subtheme encompasses organisational challenges at both the Trust (i.e., a Trust is an organisational unit within the NHS serving a geographical area) and commissioning levels. At a Trust level, MDT members described logistical and administrative challenges that hindered effective service delivery. Administrative challenges included not having the correct access to IT systems, and/or incompatible IT systems between services, and difficulties identifying relevant Human Resources processes. Some participants perceived these difficulties to be due to the use of dual contracts and/or different Trusts/organisations (e.g., contracted youth services) working together to deliver CEW services.

One of the main logistical issues cited by participants was the difficulty in accessing appropriate clinic space to meet patients. Participants believed that this impacted their ability to deliver personalised care, be that through limiting opportunities for 1:1s with patients, or to host a full in-person MDT “*for eight people in a room that only fits three*” (SL18_4):

*[Trust name] is terrible for getting clinic time and rooms*,* we really battle with that. And sometimes we don’t even get offered a second room for our clinics at [Trust name] so it’s very difficult to have that space to have kind of one to ones with the patients* (SL18_2)

At a commissioning level, while most services appeared to appreciate the flexibility given to them when setting up the service, some noted that they would have preferred more time and guidance before launch. Additionally, the level of data recording and reporting required by NHS England was often underestimated, as mentioned by this service:

*Originally*,* I don’t think there was a good understanding of how much data would be required or the consistency of data*,* so the dashboard took two or three years to develop. So*,* a lot of us had used money on clinical staff rather than admin or data staff* (SL15_1)

##### Subtheme 3.3: High demand, low capacity

This subtheme is linked to the aforementioned recruitment and organisational challenges, given that many services were not fully staffed and therefore perceived to not be working optimally. Almost all participants noted that their service had high demand that could not be met with the current capacity of the service:

*I didn’t realise that the demand was so high that we can’t physically or possibly do this in a timely manner with the current resources that we’ve got. We cannot see as many patients as we would wish to see. And then we have more referrals coming in* (SL20_3)

Participants also cited long waiting lists for specialist psychological support, such as CAMHS and eating disorder services, perceiving this may be a result of “*eating disorder services favouring underweight rather than overweight [patients]*” (SL15_3). Others reflected on instances where the complexities of CYP had deteriorated while being on waiting lists, and those who were not initially eligible for the service becoming eligible as complications worsened due to a lack of intervention:

*However*,* due to capacity*,* some of our patients have sat on a waiting list for a couple of years and in that time their complications have worsened* (SL6_2)

Participants reflected on how the lack of capacity to meet the high demand for services resulted in unplanned adaptations to the service, such as the introduction of more stringent eligibility criteria to manage demand (e.g., higher BMI SDS). Some participants also spoke about a drive to be time efficient that resulted in areas of the service being under-developed or less attended to.

*[…] something has to lose out*,* doesn’t it? So*,* if I’m doing this*,* spending this much time doing data*,* then the wait list for them to start GLP-1s has to go longer or I don’t go to a clinic on a Monday or…actual clinical work is what loses out …* (SL9_6)

## Discussion

This paper provides insights into the realities of delivering a CEW service, including the perceived challenges, and facilitators of success. The main themes revealed (1) how person-centred care was achieved in CEW services (2) how the complexity of the patient cohort impacted the CYP’s journey moving through CEW services, and (3) the challenges of designing and delivering a pilot, including recruitment challenges.

Central to the CEW programme is the use of person-centred care [23, 24]. Our findings demonstrate multiple approaches used to achieve this, and how these approaches have enabled effective care delivery. The interviews highlighted that CEW services had to build relationships with the wider hospital, community services, and schools to facilitate inter-organisational communication and support the delivery of person-centred care, which is a known facilitator of inter-organisational collaboration [25]. This approach aligns with the NHS 10 Year Health Plan ambition to shift care out of hospitals and into community-based settings through a Neighbourhood Health model [26] and reflects calls for integrated community-embedded solutions for people living with complex, chronic symptoms [27]. However, we found that the ability to forge these relationships was dependent on hospitals’ existing partnerships, personal characteristics of MDT members, and existing social capital of MDT members to spearhead this joined-up approach, which has been reported elsewhere [28]. Similarly, the interviews highlighted the importance of knowledge exchange between services to share evidence of best practice, particularly given the complexity of needs reported within the patient cohort. This reflects wider evidence on the value of inter-organisational learning within healthcare [29], underscoring the need for greater collaboration across services, particularly with leadership from more established services, to support the delivery of effective specialist weight management services for CYP.

Multidisciplinary teams represent another component of CEW services that enable person-centred care. MDTs are well established in the management of other complex paediatric conditions such as diabetes [30], cystic fibrosis [31], and kidney disease [32, 33] and more recently in the management of childhood obesity [34, 35]. Our interviews revealed that the composition of professionals in the MDT was not homogenous across CEW services (aside from core clinical roles, i.e., medical consultant and nurse), but certain competencies were considered essential to the delivery, particularly in relation to safeguarding expertise, psychological support, and family support. This finding supports a shift towards competency-based MDTs, similar to those adopted in mental health services [36], to better support the complex patient group, whilst also addressing role-specific recruitment challenges [36, 37]. Importantly, our findings reiterate that effective MDT functioning depends on appropriate IT infrastructure, adequate workspace, and sufficient administrative support [38].

While pilot programmes provide opportunities to develop innovative working practices [39], our interviews highlighted perceived job insecurity and its impact on staff recruitment and retention. This was compounded by a national shortage of mental health professionals [40], limiting some CEW services’ ability to deliver the biopsychosocial model of care that underpins the CEW programme. Nevertheless, these challenges are not unique to CEW [41], and broader efforts to improve staff retention in the NHS are ongoing [42]. Although the NHS Long Term Plan committed to treating 1,000 children per year living with severe obesity [15], CEW services are experiencing a higher demand than commissioned capacity can support (Marwood et al., [43]). Our findings suggest that these demand and capacity constraints are further exacerbated by recruitment challenges, partly linked to the short-term nature of the CEW pilot programme. Adequate long-term funding and commissioning are therefore essential to establish a permanent and fully fledged MDT to ensure service sustainability and high standards of care.

The CEW programme treats a cohort with high levels of complexity, including high levels of deprivation, neurodiversity [43], and safeguarding issues. Indeed, our data builds on this to suggest that CYP who use CEW services need long-term, flexible, holistic, person-centred support to address multiple clinical and social barriers to weight management, consistent with previous findings [24]. This flexibility includes the mode of delivery used, with both online and face-to-face remaining important components of care. As such, the NHS Long-Term Plan to reduce face-to-face outpatient appointments by a third [15] may be misaligned with the needs of the CEW patient cohort.

Furthermore, our data suggest that MDT workplans should include increased protected time dedicated to continuing professional development, to enable the continued development of skills needed to respond effectively to the complex patient needs in CEW, beyond what is offered currently. In addition, given the high emotional toll of working with clinically and socially complex groups evidenced within our data and other samples [44], it is important that MDT members continue to receive appropriate supervision and wellbeing support. Overall, long-term commissioning of the CEW programme should account for the complexity of the patient cohort, as well as the time and workforce development required to equip MDT members to deliver appropriate and efficient care.

Another key finding of this study related to the lack of appropriate long-term care for CYP accessing CEW services, including a dearth of services for CYP to transition to. This reflects both a national shortage of paediatric weight management services [45] and services to support transition into adult care. Those who are 16–18-year-old are therefore often left devoid of weight management support, which mirrors the views of the ENHANCE Patient and Public Involvement group, the ARROWs [46]. Given the evidence showing significant weight regain after paediatric weight management interventions [47] and after the cessation of weight loss medications [48], there is a need for long-term support for CYP and their families post-CEW, including appropriate wraparound and transitional services.

### Recommendations

This study has several recommendations. Firstly, there should be greater consideration for wraparound step-down and appropriate transitional adult services post-CEW. Secondly, the level of patient complexity in CYP with severe obesity needs to be anticipated to ensure appropriate and efficient care, specifically through a dedicated specialised weight management service for this population group, as Tier 2 services are unlikely to be able to provide this level of support. Thirdly, a long-term, adequately funded commissioning plan to enable the recruitment of a permanent and ‘fully-fledged’ MDT would be needed to ensure service sustainability. Fourthly, greater collaborative working between CEW services, led by those more established, would be necessary to support newer services during the design and implementation phase. This could include sharing best practice regarding logistics, workforce recruitment, and delivery models. In addition, stronger neighbourhood-based approaches to care are needed, with closer collaboration between CEW services, schools, and community organisations, recognising the social and ‘family’ nature of the condition and the limits of specialist services alone. Fifthly, to more effectively respond to the levels of patient complexity, services would need to prioritise the upskilling of the MDT in these areas, beyond what is currently provided, supported by adequate funding to do so. Finally, in parallel and recognising the demands of working with a complex and vulnerable patient group, staff wellbeing support should be strengthened and more clearly signposted.

### Strengths and limitations

The current study was co-produced with policy partners at NHSE, clinical co-investigators, and with the wider ENHANCE team, integrating expertise from policy, economics, and health, strengthening its methodological and contextual rigour. In addition, data were collected from a diverse range of professional roles within the CEW services MDT, ensuring multiple perspectives from a geographically diverse sample within England. That said, not all MDT roles were interviewed, potentially restricting the diversity of professional perspectives. In a similar vein, only MDT staff from the CEW service were interviewed, reflecting the pragmatic focus of the study on the service in which care was delivered, rather than a broader range of stakeholders involved in CYP’s care (e.g., GPs, schools). Consequently, interactions between the CEW service and the broader ‘team around the child’ are not captured. Similarly, derived themes were drawn from seven of 38 CEW services and so may not fully reflect the practices or perspectives of the whole CEW programme. It should also be noted that services included in this study were purposively selected for their innovative approaches to delivering their CEW service. This potentially may have introduced sampling bias and thus limit the generalisability of findings to less-developed services. Therefore, it is important that future commissioning decisions be taken within the context of the broader findings of the ENHANCE evaluation. Notably, patients’ lived experiences of CEW services were not captured within the current study as other research from the ENHANCE evaluation will explore this (NIHR, 158453).

## Conclusion

This study presents novel insights from MDT members on the delivery of the first NHS commissioned specialist weight management pilot for CYP in England. It highlights the distinct challenges and facilitators associated with designing and implementing a pilot programme, as well as the impact on service delivery. The findings also underscore the complexity within the CEW patient cohort, and the corresponding need for person-centred, holistic, and flexible models of care. Collectively, these findings suggest that the CEW programme represents a distinctive and innovative approach to specialist paediatric obesity service delivery, and that any future roll out could incorporate the ENHANCE evaluation recommendations to optimise models of care.

## Supplementary Information

Below is the link to the electronic supplementary material.

Supplementary Material 1

Supplementary Material 2

Supplementary Material 3

## Data Availability

The data that support the findings of this study are not openly available due to reasons of sensitivity and are available from the corresponding author upon reasonable request.

## References

[1] World Health Organization. Obesity and Overweight. 2025. Accessed March 17, 2026. https://www.who.int/news-room/fact-sheets/detail/obesity-and-overweight.

[2] Department of Health & Social Care. National Child Measurement Programme Annual Report, Academic Year 2024 to 2025. 2025. Accessed March 17, 2026. https://fingertips.phe.org.uk/static-reports/obesity-physical-activity-nutrition/national-child-measurement-programme-2024-2025-academic-year.html.

[3] An R, Yan H, Shi X, Yang Y. Childhood obesity and school absenteeism: a systematic review and meta-analysis. Obes Rev. 2017;18(12):1412–24. 10.1111/obr.12599. 28925105 10.1111/obr.12599

[4] Hunter E, Stone RA, Brown A, et al. "We go hunting …": understanding experiences of people living with obesity and food insecurity when shopping for food in the supermarket to meet their weight related goals. Appetite. 2025;205:107794. 10.1016/j.appet.2024.107794. 10.1016/j.appet.2024.107794 39615842

[5] Stiebahl S. Obesity statistics. 2025. Accessed March 17, 2026. https://commonslibrary.parliament.uk/research-briefings/sn03336/.

[6] Jebeile H, Kelly AS, O’Malley G, Baur LA. Obesity in children and adolescents: epidemiology, causes, assessment, and management. Lancet Diabetes Endocrinol. 2022;10(5):351–65. 10.1016/S2213-8587(22)00047-X. 35248172 10.1016/S2213-8587(22)00047-X PMC9831747

[7] Ward ZJ, Long MW, Resch SC, Giles CM, Cradock AL, Gortmaker SL. Simulation of Growth Trajectories of Childhood Obesity into Adulthood. N Engl J Med. 2017;377(22):2145–53. 10.1056/NEJMoa1703860. 29171811 10.1056/NEJMoa1703860 PMC9036858

[8] Daniels SR. Complications of obesity in children and adolescents. Int J Obes. 2009;33(S1):S60–5. 10.1038/ijo.2009.20. 10.1038/ijo.2009.20 19363511

[9] Hawton K, Apperley L, Parkinson J, et al. Complications of excess weight seen in two tier 3 paediatric weight management services: an observational study. Arch Dis Child. 2025;110(3):216–20. 10.1136/archdischild-2024-327286. 39477360 10.1136/archdischild-2024-327286

[10] Park MH, Sovio U, Viner RM, Hardy RJ, Kinra S. Overweight in Childhood, Adolescence and Adulthood and Cardiovascular Risk in Later Life: Pooled Analysis of Three British Birth Cohorts. PLoS ONE. 2013;8(7):e70684. 10.1371/journal.pone.0070684. 23894679 10.1371/journal.pone.0070684 PMC3722162

[11] Wiedemann UCH, van den Akker ELT, Barber TM, et al. Early-Onset of Obesity Model: Impact of Early-Onset Obesity on Comorbidity Risk and Life Expectancy. *Obes Facts*. Published online November. 2025;14:1–21. 10.1159/000549499. 10.1159/000549499 PMC12695130 41237076

[12] Förster LJ, Vogel M, Stein R, et al. Mental health in children and adolescents with overweight or obesity. BMC Public Health. 2023;23(1):135. 10.1186/s12889-023-15032-z. 36658514 10.1186/s12889-023-15032-z PMC9849834

[13] Viner RM, Kinra S, Nicholls D, et al. Burden of child and adolescent obesity on health services in England. Arch Dis Child. 2018;103(3):247–54. 10.1136/archdischild-2017-313009. 28765261 10.1136/archdischild-2017-313009

[14] Coulton V, Dodhia S, Ells L, Blackshaw J, Tedstone A. National mapping of weight management services: provision of tier 2 and tier 3 services in England. Published online 2015. Accessed March 17, 2026. https://research.tees.ac.uk/en/publications/national-mapping-of-weight-management-services-provision-of-tier-/ .

[15] NHS. The NHS Long Term Plan. 2019. Accessed September 2, 2025. https://webarchive.nationalarchives.gov.uk/ukgwa/20230418155402/https:/www.longtermplan.nhs.uk/publication/nhs-long-term-plan/.

[16] NHS England. Complications from Excess Weight (CEW) Clinics for Children. Accessed March 17. 2026. https://www.england.nhs.uk/get-involved/cyp/specialist-clinics-for-children-and-young-people-living-with-obesity/.

[17] ENHANCE. ENHANCE - evaluating the nhs england complications of excess weight clinics for children and young people (NIHR158453). 2024. Accessed March 26, 2026. https://njl-admin.nihr.ac.uk/document/download/2049203.

[18] Tong A, Sainsbury P, Craig J. Consolidated criteria for reporting qualitative research (COREQ): a 32-item checklist for interviews and focus groups. Int J Qual Health Care. 2007;19(6):349–57. 10.1093/intqhc/mzm042. 17872937 10.1093/intqhc/mzm042

[19] Heggie L, Mackenzie RM, Ells LJ, Simpson SA, Logue J. Tackling reporting issues and variation in behavioural weight management interventions: design and piloting of the standardized reporting of adult behavioural weight management interventions to aid evaluation (STAR-LITE) template. Clin Obes. 2020;10(5). 10.1111/cob.12390. 10.1111/cob.12390 32632970

[20] Olmos-Vega FM, Stalmeijer RE, Varpio L, Kahlke R. A practical guide to reflexivity in qualitative research: AMEE Guide 149. Med Teach. 2023;45(3):241–51. 10.1080/0142159X.2022.2057287. 10.1080/0142159X.2022.2057287 35389310

[21] Gale NK, Heath G, Cameron E, Rashid S, Redwood S. Using the framework method for the analysis of qualitative data in multi-disciplinary health research. BMC Med Res Methodol. 2013;13(1):117. 10.1186/1471-2288-13-117. 24047204 10.1186/1471-2288-13-117 PMC3848812

[22] Nobles J, Thomas C, Banks Gross Z, et al. Let’s Talk about Physical Activity: Understanding the Preferences of Under-Served Communities when Messaging Physical Activity Guidelines to the Public. Int J Environ Res Public Health. 2020;17(8):2782. 10.3390/ijerph17082782. 32316591 10.3390/ijerph17082782 PMC7215851

[23] Ells LJ, Homer C, Matu J, Aswani D, Steele C. Compassionate child obesity care within a stigmatising society. Lancet. 2025;406(10502):444. 10.1016/S0140-6736(25)01121-3. 40752904 10.1016/S0140-6736(25)01121-3

[24] Ells LJ, Ashton M, Li R, et al. Can We Deliver Person-Centred Obesity Care Across the Globe? Curr Obes Rep. 2022;11(4):350–5. 10.1007/s13679-022-00489-7. 36272056 10.1007/s13679-022-00489-7 PMC9589792

[25] Liapi F, Chater AM, Randhawa G, Stephenson A, Pappas Y. Factors that influence inter-organisational integration: a qualitative exploration of service providers’ perspectives from an integrated care initiative. BMC Health Serv Res. 2025;25(1):947. 10.1186/s12913-025-13051-7. 40640843 10.1186/s12913-025-13051-7 PMC12247228

[26] NHS. Fit for the Future: 10 Year Health Plan for England. 2025. Accessed September 2, 2025. https://www.gov.uk/government/publications/10-year-health-plan-for-england-fit-for-the-future/fit-for-the-future-10-year-health-plan-for-england-executive-summary.

[27] Barker C. Long-term conditions and symptom-based disorders – A community perspective. Future Healthc J. 2026;13(1):100499. 10.1016/j.fhj.2026.100499. 41704294 10.1016/j.fhj.2026.100499 PMC12907643

[28] Martin GP, Finn R, Currie G. National evaluation of NHS genetics service investments: emerging issues from the cancer genetics pilots. Fam Cancer. 2007;6(2):257–63. 10.1007/s10689-007-9130-3. 17520352 10.1007/s10689-007-9130-3

[29] Aunger JA, Millar R, Greenhalgh J, Mannion R, Rafferty AM, McLeod H. Why do some inter-organisational collaborations in healthcare work when others do not? A realist review. Syst Rev. 2021;10(1):82. 10.1186/s13643-021-01630-8. 33752755 10.1186/s13643-021-01630-8 PMC7984506

[30] Swallow V, Horsman J, Mazlan E, et al. DigiBete, a Novel Chatbot to Support Transition to Adult Care of Young People/Young Adults With Type 1 Diabetes Mellitus: Outcomes From a Prospective, Multimethod, Nonrandomized Feasibility and Acceptability Study. JMIR Diabetes. 2025;10:e74032–74032. 10.2196/74032. 40699892 10.2196/74032 PMC12309419

[31] Frost F, Dyce P, Ochota A, et al. Cystic fibrosis-related diabetes: optimizing care with a multidisciplinary approach. Diabetes Metab Syndr Obes. 2019;12:545–52. 10.2147/DMSO.S180597. 31118718 10.2147/DMSO.S180597 PMC6499442

[32] Nightingale R, Sinha MD, Swallow V. Using focused ethnography in paediatric settings to explore professionals’ and parents’ attitudes towards expertise in managing chronic kidney disease stage 3–5. BMC Health Serv Res. 2014;14(1):403. 10.1186/1472-6963-14-403. 25234741 10.1186/1472-6963-14-403 PMC4176584

[33] Swallow V, Smith T, Webb NJA, et al. Distributed expertise: qualitative study of a < scp>B ritish network of multidisciplinary teams supporting parents of children with chronic kidney disease. Child Care Health Dev. 2015;41(1):67–75. 10.1111/cch.12141. 24827413 10.1111/cch.12141 PMC4368419

[34] Tee LP, Brandreth RA, Sauven N, Clarke L, Frampton I. Successful outcomes in childrens specialist weight management: Impact assessment of a novel early years weight management programme. J Hum Nutr Dietetics. 2021;34(5):819–26. 10.1111/jhn.12872. 10.1111/jhn.12872 33894093

[35] Lanigan J, Sauven N. Treatment of childhood obesity: a multidisciplinary approach. Clin Integr Care. 2020;3:100026. 10.1016/j.intcar.2020.100026.

[36] Kutcher S, Davidson S, Manion I. Child and youth mental health: Integrated health care using contemporary competency-based teams. Paediatr Child Health. 2009;14(5):315–8. 10.1093/pch/14.5.315. 20436824 10.1093/pch/14.5.315 PMC2706634

[37] Meadows D, Maclaren J, Morton A, Ross D. Determining skill mix and optimal multidisciplinary team composition: A scoping review. Healthc Manage Forum. 2025;38(3):278–85. 10.1177/08404704241293095. 39498670 10.1177/08404704241293095 PMC12009454

[38] Pradelli L, Risoli C, Summer E, et al. Healthcare professional perspective on barriers and facilitators of multidisciplinary team working in acute care setting: a systematic review and meta-synthesis. BMJ Open. 2025;15(3):e087268. 10.1136/bmjopen-2024-087268. 40118478 10.1136/bmjopen-2024-087268 PMC11931918

[39] Goff M, Hodgson D, Bailey S, Bresnen M, Elvey R, Checkland K. Ambiguous workarounds in policy piloting in the NHS: Tensions, trade-offs and legacies of organisational change projects. New Technol Work Employ. 2021;36(1):17–43. 10.1111/ntwe.12190.

[40] British Medical Association. Measuring progress: commitments to support and expand the mental health workforce in England. 2020. Accessed March 17, 2026. https://www.bma.org.uk/media/2405/bma-measuring-progress-of-commitments-for-mental-health-workforce-jan-2020.pdf.

[41] Teo H, Vadean F, Saloniki EC. Recruitment, retention and employment growth in the long-term care sector in England. Front Public Health. 2022;10. 10.3389/fpubh.2022.969098. 10.3389/fpubh.2022.969098 PMC9650477 36388378

[42] NHS England. NHS retention drive expanded across the country with thousands fewer staff leaving frontline roles. 2023. Accessed March 17, 2026. https://www.england.nhs.uk/2023/12/nhs-retention-drive-expanded-across-the-country-with-thousands-fewer-staff-leaving-frontline-roles/.

[43] https://karger.com/ofa/articlepdf/doi/10.1159/000551826/4536238/000551826.pdf.

[44] Shaw RL, Butcher I, Webb S, Duncan HP, Morrison R. Building evidence-based interventions to improve staff well‐being in paediatric critical care using the behaviour change wheel. Nurs Crit Care. 2025;30(4). 10.1111/nicc.13228. 10.1111/nicc.13228 PMC12210092 39780506

[45] Mears R, Leadbetter S, Candler T, Sutton H, Sharp D, Shield JPH. Cross-sectional survey of child weight management service provision by acute NHS trusts across England in 2020/2021. BMJ Open. 2022;12(11):e061971. 10.1136/bmjopen-2022-061971. 36356995 10.1136/bmjopen-2022-061971 PMC9670955

[46] ENHANCE. ENHANCE ARROWS. 2024. Accessed March 17, 2026. https://www.enhance-research.com/enhance-arrows.

[47] Vermeiren E, Bruyndonckx L, De Winter B, Verhulst S, Van Eyck A, Van Hoorenbeeck K. The effect of weight regain on cardiometabolic health in children with obesity: A systematic review of clinical studies. Nutr Metabolism Cardiovasc Dis. 2021;31(9):2575–86. 10.1016/j.numecd.2021.05.020. 10.1016/j.numecd.2021.05.020 34172320

[48] West S, Scragg J, Aveyard P, et al. Weight regain after cessation of medication for weight management: systematic review and meta-analysis. BMJ. 2026;392:e085304. 10.1136/bmj-2025-085304. 41500720 10.1136/bmj-2025-085304 PMC12776922

