# Supplementary material for: “It’s the biggest not one-size-fits-all service I’ve ever worked in”: the realities of delivering a ‘Complications of Excess Weight’ service for children and young people in England from a multidisciplinary team perspective
Source: BMC Health Serv Res. 2026 Jun 5;26:819. doi: 10.1186/s12913-026-14899-z (PMC13270842; doi:10.1186/s12913-026-14899-z)
Supplement: Supplementary file 1 — Supplementary Material 1 [file 12913_2026_14899_MOESM1_ESM.docx]

**Evaluating the NHs englANd Complications from Excess weight clinics for children and young people: ENHANCE**

**CEW Clinic Adaptations Interviews (WP2.3)**

| **Introduction and audio consent**   - *Introduction* – Introduce self - *Aim* – NHS England have recently invested in the Complications from Excess Weight (CEW) Clinics. These are specialist clinics for children living with severe and complex forms of obesity, with a view to preventing the onset of future co-morbidities such as type 2 diabetes, cardiovascular diseases, and poor psychosocial health, and treating and reversing co-morbidities which are already present. The ENHANCE project will evaluate the design and delivery of the CEW clinics which are being rolled out across England.   Guided by the findings from their STAR-LITE survey responses, stakeholders from a selection of CEW clinics have been invited to an online interview to understand the reality of designing and delivering their CEW clinic.   - *Consent* – explain voluntary participation, audio record, anonymous quotes - Switch audio recorder on - For the audio recording, can I check again that: - You have had the opportunity to ask any questions about the study and have them answered. - You agree to take part in the study?   (n.b. participants will previously have provided written consent) |
| --- |

|  | |
| --- | --- |
| **1.** | **Involvement** \| What is your role in the CEW clinic and how long have you been involved in the clinic? |
| **2.** | **Delivery of CEW** \| We’d like to understand more about your experiences of delivering a CEW clinic. What has this been like from your perspective?   - *Probe*: How has demand and capacity compared to what you expected in your CEW clinic? - *Probe*: Can you share your experiences of working with an MDT in the clinic, and what factors have influenced that experience? - *Probe*: Have you had to adapt your services since the beginning. What were the reasons for this? - *Probe*: Has there been much engagement with the CEW clinic network? - TAILORED Q: We've noticed that your service includes children starting at 7-years of age, which is older than other services. Could you tell us more about this and your experience of this? [note – you could bring this up when you discuss ‘age’ in question 3?] |
| **3.** | **Patient group** \| How has the patient cohort compared to what you expected?   - Prompt: Considering things like complexity, age, neurodiversity, demographics, and poverty? - *Probe*: Have you had to adapt services in response to the unanticipated needs of the patient cohort? |
| **4.** | **Fit with wider weight management system** \| How does your CEW clinic connect with other local weight management services, such as Tier 2 and Tier 4 services? |
| **5.** | **Enablers** \| What other factors have made it easier to deliver your CEW clinic?   - *Prompt*: For example, dedicated admin support, links to community services, or space for MDT working? |
| **6.** | **Further challenges** \| Have there been any other challenges we have not already discussed regarding the delivery of a CEW clinic? |
| **7.** | **What does good look like \|** From your perspective, now that the service has been running for a while, what would your ideal CEW clinic look like and what would be needed to get there? |
| **Specific questions relating to learning from the ENHANCE evaluation** | |
| **8.** | **(If relevant) Safeguarding** \| Another point coming from the wider evaluation relates to safeguarding concerns and how to efficiently manage these.  What’s your experience with safeguarding concerns in your clinic, and how do you currently manage them? |
| **9.** | **(If relevant) Transition / onward services** \| Another observation from the evaluation relates to the processes and criteria for discharging patients or transitioning them into onward services. What has your experience been of this? [note – if they start talking about Engagement/DidNotAttend/WasNotBrought, ask how this could have been improved or what impacted this?   - *Probe*: Is there provision for 16-18 year-olds in your service. If yes, can they then access adult weight management upon discharge? If not, why is there no provision here? - *Probe*: Can patients re-refer into your CEW service and what does this process look like locally? |
| **Closing remarks** | |
| **10.** | **Anything else** \| Is there anything else that you would like to mention regarding your experiences of delivering a CEW clinic? |

THANK PARTICIPANTS FOR THEIR TIME.
