# Supplementary material for: “It’s the biggest not one-size-fits-all service I’ve ever worked in”: the realities of delivering a ‘Complications of Excess Weight’ service for children and young people in England from a multidisciplinary team perspective
Source: BMC Health Serv Res. 2026 Jun 5;26:819. doi: 10.1186/s12913-026-14899-z (PMC13270842; doi:10.1186/s12913-026-14899-z)
Supplement: Supplementary file 2 — Supplementary Material 2 [file 12913_2026_14899_MOESM2_ESM.docx]

Supplementary File 2 – Researcher Positionality Statements

**RAS** is a White British female Early Career Researcher with a PhD in the psychology of children’s eating behaviour. RAS is a mixed methods researcher and has worked on previous public health evaluations, such as reviewing the High Fat Sugar and Salt (HFSS) policy on retail purchase patterns (DIO Food project). RAS led the design, recruitment, and data collection of the current research and dually led the analysis of the interview data with JMa. RAS is currently working as a Research Fellow in the Obesity Institute at Leeds Beckett University.

**JMa** is a White British female Early Career Researcher with a PhD in the psychology of binge eating. JMa is a mixed methods researcher and has worked on previous public health evaluations including the review of the NHS Total Dietary Replacement programme for Type 2 Diabetes. JMa dually led the analysis of the interview data with RA. JMa is currently working as a Research Fellow in the Obesity Institute at Leeds Beckett University

**PD** is a White British female Early Career Researcher with a PhD in Health Promotion. PD is a mixed methods researcher and has worked on previous public health research and evaluations at Leeds Beckett University. PD conducted a proportion of the interviews and contributed towards the writing of the methods section. PD previously worked in the Obesity Institute at Leeds Beckett University and is now working in The School of Health – Health Promotion.

**JN** is a white British male mid-career researcher with a PhD in childhood weight management. He is a mixed methods researcher with 10+ years working in applied public health, both evaluating interventions (e.g. active travel schemes, unhealthy advertising reduction policies, systems approaches) and in developing methods to support intervention design, delivery and evaluation (e.g. Action Scales Model, Ripple Effects Mapping, Public Health England Whole Systems Approach to Obesity guidance). JN is work-package lead in the ENHANCE evaluation and as such supported conceptualisation, planning and delivery of the current study. JN is currently a Reader of Public Health at Leeds Beckett University.
