# Supplementary material for: “It’s the biggest not one-size-fits-all service I’ve ever worked in”: the realities of delivering a ‘Complications of Excess Weight’ service for children and young people in England from a multidisciplinary team perspective
Source: BMC Health Serv Res. 2026 Jun 5;26:819. doi: 10.1186/s12913-026-14899-z (PMC13270842; doi:10.1186/s12913-026-14899-z)
Supplement: Supplementary file 3 — Supplementary Material 3 [file 12913_2026_14899_MOESM3_ESM.docx]

Supplementary File 3

**Theme 1: The challenges of designing and delivering a pilot service**

***Subtheme 1.1: Recruiting and retaining staff***

*I think it's something that affects all of us because, service is new and most of the staff who are in post, they just don't know about their future. They don't know about their future because you could, come March or the next funding is agreed and then you don't have a role. So that just brings some anxiety in, in members of staff. And I think maybe in the past people have just decided I want to go and look for something more secure because I don't know where I stand in terms of my role because it's quite temporary. So, I think that's really affecting 'cause you, you’re missing good talent.*(SL20_3)

*We did have an advanced nurse practitioner working in the team to manage the medication. That was the big thing. So, we set up medication weight loss injection clinics with her. So, to manage the dose increases and also because she was a prescriber. So that was great. Because I'm the only clinician in the team, it is a bit difficult sometimes if I'm busy doing other, you know, ward work or on leave then there is no other clinician. So, managing medications is difficult. So that was good, but she's left the service now so we're now without a medication clinic, but that's what we would like to re-set up.*(SL13_1)

***Subtheme 1.2: Organisational challenges***

*[Trust name] and [Trust name] had never worked together before. So, they put in this joint bid. And obviously they, they won the bid and had this joint CEW. But there, there was no sort of contract collaboration or anything like that, so I had to do all of that from scratch... But it took months to get accesses, contracts, just basic IT things. We've still got members of staff that can't see notes on the side that isn't paying their money*(SL18_4)

***Subtheme 1.3: High demand, low capacity***

*I think having been here for the last eight months, I didn't realise that the demand was so high that we can't physically or possibly do this in a timely manner with the current resources that we've got. So, our waiting list now goes to nine months. Which, which is quite a lot. And it's increasing because we are not able to see as many patients as we would, we would wish to see. And then we have more referrals coming in. (SL20_3)*

**Theme 2: Navigating complexity in the patient journey**

***Subtheme 2.1: Describing and responding to complexity***

*“I think the team have also had another [safeguarding case] which has taken up a lot of thought and time. So maybe two cases that I can think of where there's been kind of serious safeguarding concerns. I think it's a challenge though, because where does sort of a parent’s approach to feeding their child/helping their child access exercise go from being slightly not ideal to neglect? And it's a fuzzy one at times”* (SL4_3)

***Subtheme 2.2: The CEW patient journey***

*“We do have a number of patients who don't engage after referral.  So that's how referrers, they [CYP] see another paediatrician who recognises their BMI is very high and says, “I want you to go to weight management”, but doesn't explain that to the patient, doesn't explain what weight management clinics entail, and doesn’t explain properly why they're worried about weight and the health impacts. So, the patient doesn't have any buy-in, but they agree because people like to say yes to their doctor, and then they just don't answer the text or the phone to CEW ever. We have quite a lot of those and we send engagement letters and then if they don't respond, we take them off the waiting list basically”*(SL13_1)

*“We could say, well look, don't worry, this person will be in contact with you or here's your go to person now. So, I often think we represent this pyramid of what services should look like, and we are really setting ourselves up and crystallising what we need to do at the top, yet there's absolutely nothing below us. So, that makes all of these things like, who do you discharge and when do you discharge, and can you discharge someone who's been with the service for three years, but whose BMI is rising? Yes, no, don't know. But if you keep them in the service and you don't have any other treatment to offer them, then what's the point? But then for the patient that's a bit like well, sorry, you've, you've failed at weight management and now we're going to discharge you”*(SL13_1)

*“I would say it takes us probably up to like 12 months to get that relationship building with our families. Some of these young people don't want to speak to us. So, a lot of the work in the beginning is around developing a relationship, building up trust, all that sort of stuff. So that takes quite a long time, and then to actually get into a phase where you're moving forward with making changes and things like that, that's, that's tough and it just takes a long time. So, actually, are they ready for discharge at two years? I'm not sure they are, to be honest. And I suppose that's impacted on by the number of patients that we have, because if we were able to see them on a weekly basis or a twice week, two weekly basis or something like that, then that would enable us to do that more intensive work. But we don't have the capacity to do that because we have such a high volume of patients”*(SL9_5)

*"This job is relentless at times, and it can feel very much like you are drowning and not making any difference […] I recently discharged a patient that was going to turn 18 the following week and she was in floods of tears and didn't want to leave and didn't know how she was going to cope without us, and why can't she stay till she's 25, and it's so unfair and she's done so well. She knows that she's going to just absolutely fall off the wagon when she leaves. And it was devastating to see that we'd reduced her BMI from 54 to 42 in the time that we'd had her. It made me tear up myself sat in the clinic room with her crying, begging us not to discharge her...You pour your heart and soul into these families, and you work with them for so long and not being able to know that they've got a little bit of a safety net after us is hard"*(SL6_2)

**Theme 3: Achieving person-centred care in CEW services**

***Subtheme 3.1: The composition and function of the MDT***

*“We've had so many young people where I think if we had a social worker embedded in the service and we could have just sought their advice from the beginning rather than having to then go to safeguarding team and see where we're going to progress things and how we're going to get on. Especially with some of the younger ones that we've had, or one of the quite tricky patients that we had when they first came to us, it would just honestly would make everyone's life so much easier. It would definitely make the family support worker’s life so much easier, she could then focus on actually doing the family support work rather than the social side" (SL18_1)*

*“And I think in terms, clinically with the money I think the one role that I feel we should have had more of is family support work […] I think these younger families are the ones that really need so much time, the parents need so much time. It's not so much the children, it's the families. There's a lot of social stuff. There's a lot of parenting. There's a lot of setting boundaries. And I think the family support worker is 100% worth her weight in gold” (SL18_1&2)*

***Subtheme 3.2: Relationships beyond the walls of the CEW service***

*“We [wider CEW network) have a monthly meeting, and we discuss challenges, what's working well, and I've got lots of resources from and I've shared a lot of resources. I think the big thing for myself, I collect a lot of feedback from young people, and I don't think other services are doing that necessarily. But again, lots of other services are doing lots of other things which I'd like to replicate. So, I've just taken things which don't cost, for example, certain worksheets or certain ideas" (SL18_3)*
